# Skull base osteomyelitis with extensive bony erosion complicated by venous sinus thrombosis

**DOI:** 10.1002/ccr3.7252

**Published:** 2023-04-25

**Authors:** Tyler J. Gathman, Autefeh S. Sajjadi, Paul D. Kleinschmidt

**Affiliations:** ^1^ School of Medicine University of Minnesota Minneapolis Minnesota USA; ^2^ Department of Otolaryngology University of Minnesota Minneapolis Minnesota USA; ^3^ Department of Medicine Veterans Affairs Health System Minneapolis Minnesota USA

**Keywords:** ear, infectious diseases, nose and throat

## Abstract

Skull base osteomyelitis (SBO) mimics the presentation of various conditions, including solid tumors. Computed tomography‐guided core biopsy for culture informs antibiotic selection, and with intravenous corticosteroids, may minimize chronic neurologic dysfunction. Although SBO predominantly affects individuals who are diabetic or immunocompromised, it is important to be able to recognize SBO presenting in an otherwise healthy individual.

## 1 INTRODUCTION

Skull base osteomyelitis (SBO) involves an inflammatory process of the temporal, sphenoid, and occipital bones that commonly presents with severe otalgia, purulent otorrhea, headache, and conductive deafness from Eustachian tube dysfunction.^1^ SBO findings confer a broad differential that includes solid tumors, granulomatosis with polyangiitis, and malignant hemopathies, thereby presenting a diagnostic dilemma. The nonspecific presentation can delay diagnosis which may grant a worse prognosis. Computed tomography (CT) and magnetic resonance imaging (MRI) are mainstays for characterizing the infectious process, and image‐guided biopsy may be required to differentiate from solid tumors and also provide pathogen cultures.^2^

Skull base osteomyelitis leads to significant morbidity with the development of meningitis, intracranial abscess, and venous sinus thrombosis.^3^ In the long term, cranial nerve dysfunction may persist indefinitely despite adequate disease management.^4^ Treatment requires an extended course of culture‐directed antibiotics and potential surgical debridement.^5^

Herein, we present the case of a previously healthy 70‐year‐old man with a constellation of unilateral otalgia, otorrhea, multiple cranial neuropathies, and severe headaches that was diagnosed with SBO, extensively eroding the skull base. The case was complicated by cerebral venous sinus thrombosis and gastrointestinal (GI) bleeding before the patient made a full recovery without chronic neurologic sequalae. The case demonstrates an unusual presentation of SBO that was promptly diagnosed with MRI imaging and CT‐guided biopsy, and managed with culture‐directed antibiotics and intravenous corticosteroids to minimize the potential for chronic neurologic dysfunction.

## 2 CASE

An otherwise healthy 70‐year‐old male with a history remarkable for 30 pack‐years of cigarette smoking presented to the emergency department (ED) with worsening right‐sided otalgia and otorrhea. The patient initially began experiencing otalgia 3 months prior to presentation after undergoing a dental procedure. The otalgia improved slightly with intermittent antibiotics but became increasingly severe, prompting ED presentation. On arrival, he also reported frontal‐to‐right temporal headaches and worsening dysphagia, initially to solids and progressing to include liquids resulting in a 20‐pound weight loss, and subsequent severe malnutrition, over the span of 3 weeks. He denied any changes to his hearing or vision.

**FIGURE 1:**
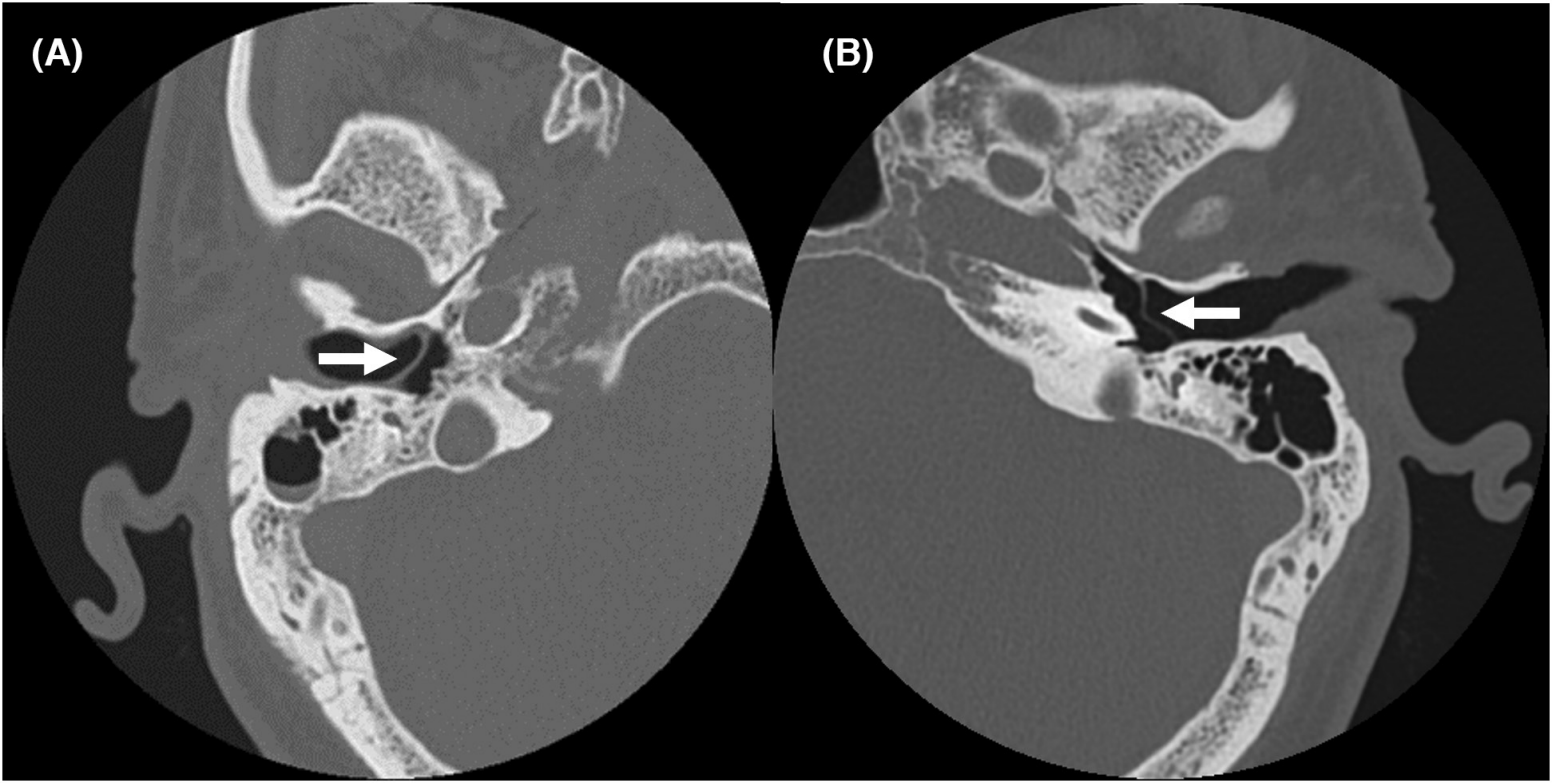
Temporal bone axial CT showing right TM (arrows) thickened with narrowed EAC (A) compared to the normal left TM and EAC (B).

On examination, the patient had a hoarse voice, right palatal weakness, and tenderness along the right lateral neck and peri‐auricular region. There was mild edema of the right external auditory canal (EAC) as well as scant otorrhea, however the right tympanic membrane (TM) was intact (Figure 1). There was no facial weakness, and the tongue was midline. He was afebrile, and labs were notable for mildly elevated white blood cell count (13.51 × 10^9^/L) and C‐reactive protein (15.34 mg/L).

**FIGURE 2:**
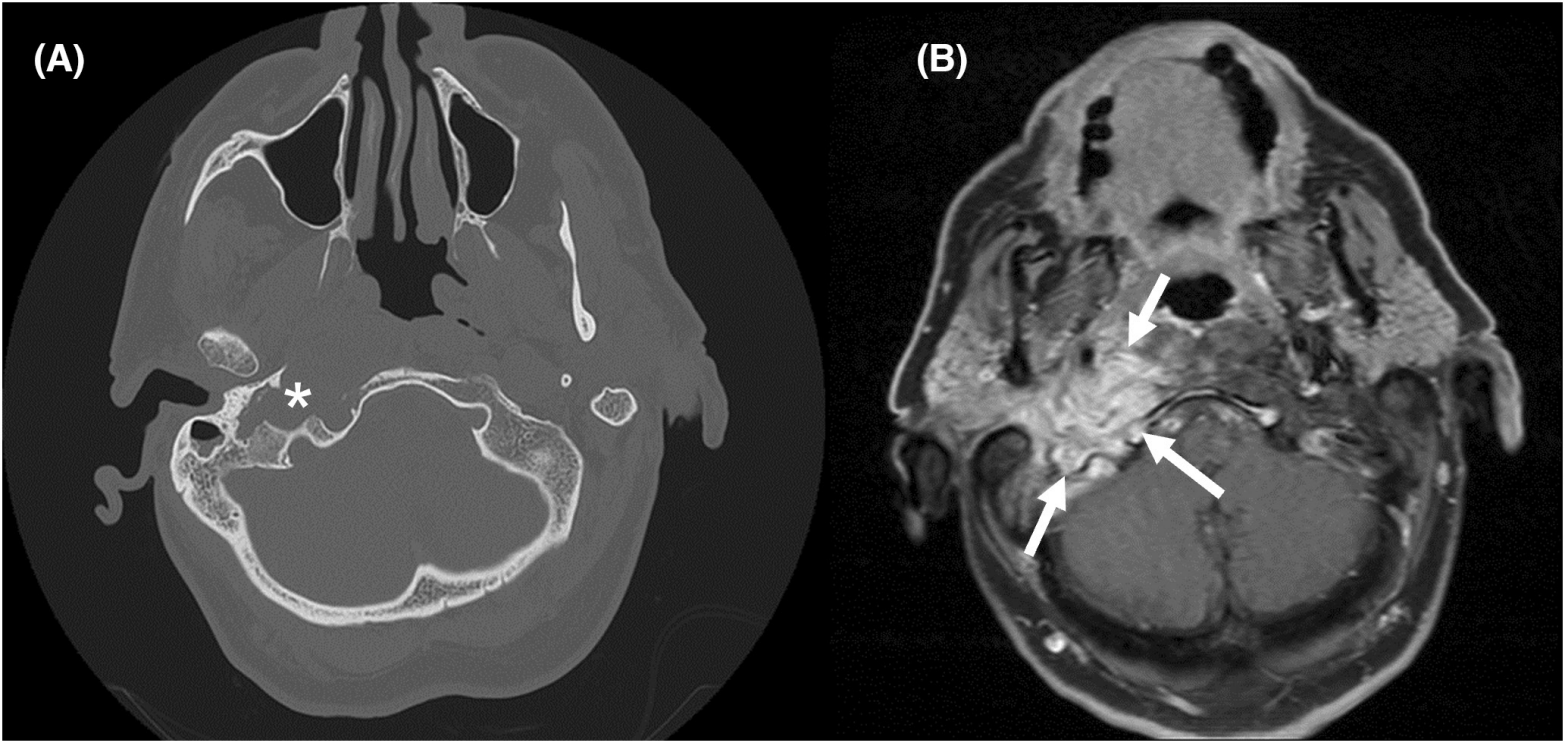
Axial CT (A) and T1 Dixon MRI (B) demonstrating right central skull base bone erosion (asterisk) with infiltrating soft tissue mass (arrows).

A head and neck CT without contrast was obtained by the ED and demonstrated a significant infiltrative process encompassing the right upper neck and skull base (Figure 2A). There was accompanying bony destruction and invasion of the occipital bone with extension to the right hypoglossal canal, jugular bulb, and stylomastoid foramen. MRI with T1 Dixon sequence demonstrated hyperintensity at the right skull base with infiltration of the occipital bone (Figure 2B). Mild meningeal enhancement and thickening were present without findings of an intracranial abscess. To evaluate dysphagia, a barium swallow study was performed that demonstrated persistent laryngeal penetration with aspiration. The patient was placed on a full‐liquid diet with aspiration precautions until swallow function improved to tolerate soft and bite‐sized diet.

**FIGURE 3:**
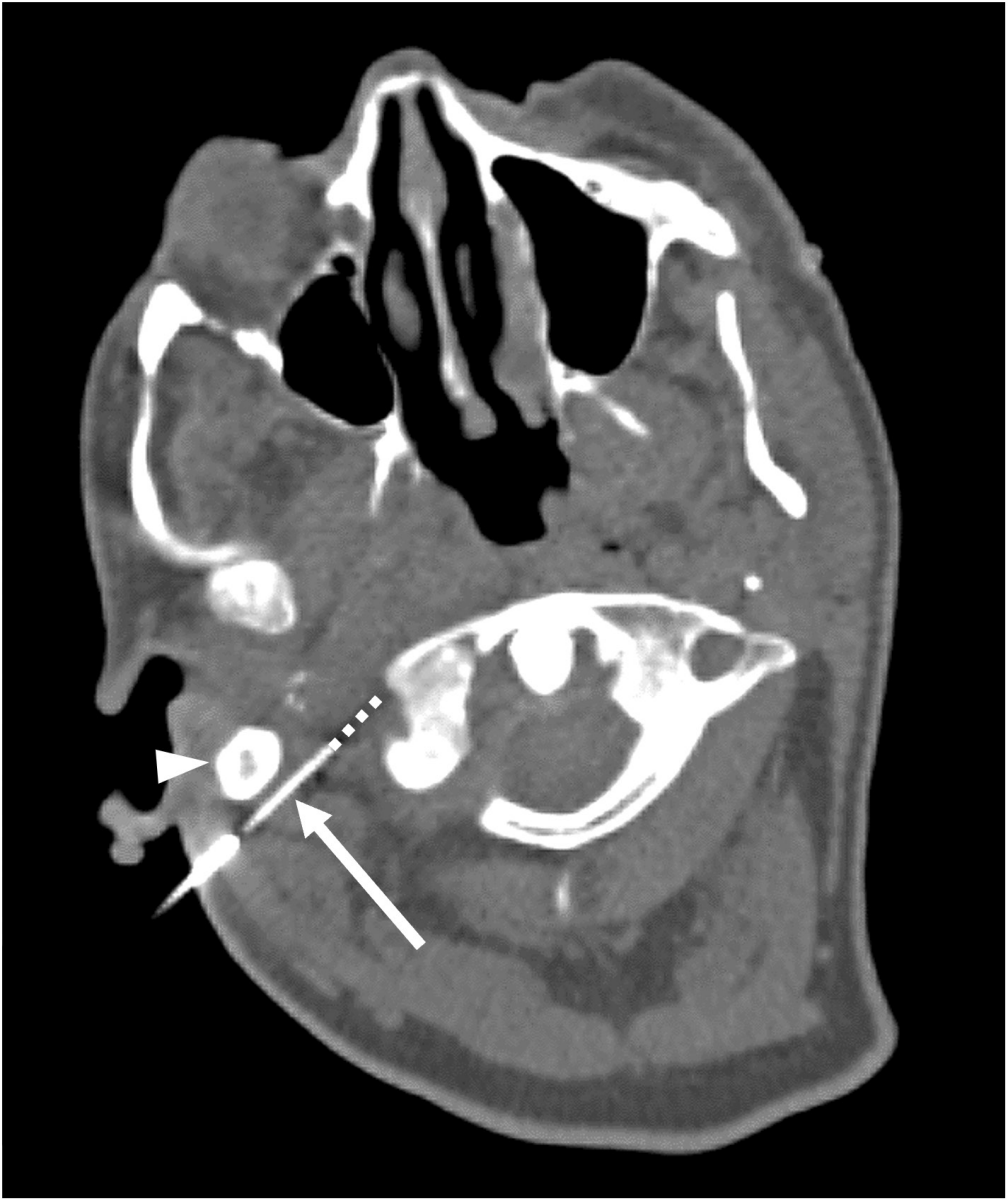
Percutaneous CT‐guided biopsy imaging demonstrating the retromastoid (wedge) course (dashed line) of core biopsy needle (arrow) to sample skull base lesion.

Imaging findings were concerning for skull base infectious process or invading malignancy. The patient was initially started on empiric intravenous vancomycin (15 mg/kg every 12 h), cefepime (2 g every 8 h), voriconazole (500 mg every 12 h), and metronidazole (1000 mg every 12 h). A CT‐guided core biopsy of the soft tissue infiltrate was obtained within 24 h of admission. Percutaneous insertion of a 20‐gauge core biopsy needle with a retromastoid approach was utilized to avoid the facial nerve course, and the occipital artery was visualized anteriorly with CT imaging (Figure 3). Pathology was negative for neoplastic processes, while aerobic culture grew pan‐sensitive *Pseudomonas aeruginosa*. The antibiotic regimen was subsequently narrowed to culture‐directed intravenous cefepime (2 g every 8 h) on inpatient day 4.

On inpatient days 3 and 4, despite treatment with anti‐Pseudomonal medication, the patient developed progressive and severe dysphonia and dysphagia with inability to tolerate the full‐liquid diet. There was concern for impending airway compromise that may necessitate endotracheal intubation. Intravenous corticosteroids (dexamethasone 6–10 mg every 8 h) with a taper were initiated. Throughout days 5 and 6, dysphonia and dysphagia were ameliorated, avoiding endotracheal intubation. Lateral neck and peri‐auricular tenderness also gradually improved.

On day 7 of hospitalization, the patient developed worsening and intractable headaches, hematemesis, and melena prompting transfer to the intensive care unit (ICU) due to hemodynamic instability. Upper GI endoscopy visualized an actively bleeding duodenal ulcer, and the patient was started on intravenous pantoprazole (40 mg daily). Due to his severe headaches, a magnetic resonance venogram was performed and identified a sigmoid and transverse sinus thrombosis. With attention to the concurrent GI bleed, the patient was cautiously initiated on low‐intensity intravenous heparin (25,000 units every 24 h), which was later transitioned to oral anticoagulation (apixaban 5 mg twice daily) without any further concerns for hemorrhage. The patient was transferred from the ICU back to inpatient medicine on day 9, and remained hospitalized for an additional week.

After the 18‐day hospitalization, the patient was safely discharged home with a peripherally inserted central catheter to continue his intravenous cefepime regimen. At discharge, cranial neuropathies, including dysphagia, palatal weakness, and hoarseness were resolved, and symptoms were limited to mild headaches and slight lateral neck and post‐auricular tenderness.

Six weeks after discharge during outpatient follow‐up, the patient demonstrated normal cranial nerve function, and resolution of the headaches and peri‐auricular tenderness. The patient was transitioned from intravenous cefepime to oral ciprofloxacin (750 mg twice daily) for 3 months with complete resolution of infection and normalization of C‐reactive protein. Oral anticoagulation with apixaban (5 mg twice daily) was continued for prophylaxis against venous thrombosis.

## 3 DISCUSSION

Skull base osteomyelitis is an uncommon condition that can lead to significant morbidity, including cranial neuropathies and intracranial complications. Cranial nerve (CN) dysfunction can frequently occur with extension to the jugular foramen (CNs IX, X, and XI), stylomastoid foramen (V), and petrous apex (V and VI) leading to dysphagia, dysphonia, and facial weakness.^1^, ^3^ Many pathogens, including *Pseudomonas*, *Streptococcus*, and fungal species, can lead to SBO.^1^, ^6^ A leading cause of SBO remains malignant otitis externa (MOE) with a *Pseudomonas* infection occurring in >90% of cases, but SBO can also occur from odontogenic, otogenic, sinogenic, and mastoiditis infections.^7^ The pathogenesis of SBO remains unclear. However, cerumen pH alternations and impaired immune response and vascular perfusion may provide the necessary environment for SBO, as evidenced by most patients being diabetic, elderly, or immunocompromised which encompass the leading risk factors for SBO.^8^

Clinically, SBO may demonstrate signs of overt infection including fever, leukocytosis, or elevated inflammatory markers. Imaging studies, commonly utilized for early diagnosis for localization and extent of infection, are often nonspecific but may demonstrate soft tissue infiltration and bony erosion of the skull base. Compared to CT, MRI has been shown to have better sensitivity for characterizing disease infiltrate, intracranial extension, and treatment response, and has been considered the Gold Standard for detecting an infectious lesion.^1^, ^9^ Nonetheless, CT has utility in assessing bony erosion of the skull base. SBO can frequently be misdiagnosed for malignancy or granulomatosis which makes biopsy with histopathology necessary. However, depending on the anatomical location and extension of the infection, obtaining a biopsy may be difficult. Percutaneous CT‐guided biopsy provides a minimally invasive, safe, and highly diagnostic method of obtaining disease infiltrate for characterization.^2^

Treatment for bacterial SBO involves an extended course of culture‐directed intravenous antibiotics for a minimum of 6 weeks to achieve complete resolution.^5^, ^10^ Surgical intervention for debridement of infected tissue may be beneficial for source control but can lead to unnecessary morbidity. If left untreated, SBO leads to significant mortality with the development of meningitis, intracranial abscess, and venous sinus thrombosis. Overall, the long‐term prognosis of SBO was reported to be 90.5% within 18 months, with 31% developing neurologic sequelae, though certain medical conditions such as diabetes have demonstrated worse prognosis.^1^, ^4^

For the patient in the present case, aside from advanced age, there were no known risk factors for SBO as hemoglobin A1c was normal (5.8%), and human immunodeficiency virus testing returned negative. It was hypothesized the SBO infection was secondary to MOE that may have been a result of odontogenic seeding with subsequent extension to the skull base. Nonetheless, *Pseudomonas* is an uncommon odontogenic infiltrate making it an unlikely presentation. Notably, in the context of deteriorating cranial nerve function due to skull base compression and inflammation, endotracheal intubation was avoided secondary to intravenous corticosteroids. The corticosteroid regimen likely reduced neuronal inflammation and compression leading to transient restoration of baseline function until antibiotic treatment could alleviate the infectious process. However, corticosteroids blunt the immune response, highlighting a potential tradeoff that should be exercised with caution in SBO. Overall, despite ICU intervention and an aggressive skull base erosive process, the patient made a full recovery without chronic neurologic sequalae.

The case underlines the importance of recognizing SBO versus similarly presenting malignancy, especially in patients lacking traditional risk factors, for prompt culture‐directed antibiotic treatment leading to a favorable outcome. Additionally, the utilization of CT‐guided biopsy for differentiation from solid neoplastic tumors and pathogen isolation was effective in promptly diagnosing SBO and narrowing empiric antibiotic treatment to cefepime. Corticosteroids may also be used judiciously in avoiding rapid decline from cranial nerve dysfunction and chronic neurological impairment.

## 4 CONCLUSION

Skull base osteomyelitis is a rare inflammatory condition that presents similarly to multiple diseases including malignancy. The condition has high morbidity and mortality with possible long‐term neurologic impairment if not promptly treated. MOE often precedes SBO with *Pseudomonas aeruginosa* being the most isolated pathogen. Patients with diabetes mellitus, advanced age, or that are immunocompromised are especially susceptible to SBO. The diagnosis often requires CT or MRI, along with image‐guided biopsy, which characterizes the disease infiltrate and presence of bony erosion. Potential complications include meningitis, intracranial abscess, and venous sinus thrombosis. Treatment involves extended‐course antibiotics, potential surgical debridement, and anticoagulation in the case of venous thrombosis.

We demonstrate that even in an unusual presentation of SBO with a broad differential, prompt culture‐directed antibiotics with corticosteroid taper can resolve neurologic dysfunction and clear infection, with a favorable long‐term prognosis. Future studies are needed to assess the utility of CT‐guided biopsy and intravenous corticosteroids in the long‐term outcomes of SBO, especially in patients where SBO may not be initially diagnosed due to lack of risk factors.

## ETHICAL APPROVAL

Exempt; Patient Permission Obtained.

## CONSENT

Written and verbal patient consent was obtained for the publication of the case report. Written consent is available upon request.

## AUTHOR CONTRIBUTIONS

**Tyler J. Gathman:** Conceptualization; data curation; investigation; writing – original draft; writing – review and editing. **Autefeh S. Sajjadi:** Conceptualization; formal analysis; investigation; writing – original draft; writing – review and editing. **Paul D. Kleinschmidt:** Conceptualization; investigation; supervision; writing – review and editing.

## CONFLICT OF INTEREST STATEMENT

None.

## FUNDING INFORMATION

The study was not funded.

## Data Availability

Data is available upon request to authors.
